# Analysis of the functional performance of amputees on the timed up and go scale compared to healthy volunteers: cross-sectional observational study

**DOI:** 10.1016/j.clinsp.2026.100881

**Published:** 2026-04-09

**Authors:** Juliana dos Santos Guilherme Ribeiro, Denise Matheus, Rafael de Albuquerque Lima, Artur Cesar Aquino dos Santos, Andre Tadeu Sugawara, Linamara Rizzo Battistella, Felipe Fregni, Marta Imamura

**Affiliations:** a Instituto de Medicina Física e Reabilitação, Hospital das Clínicas, Faculdade de Medicina, Universidade de São Paulo (HCFMUSP), São Paulo, SP, Brazil; b Departamento de Medicina Legal, Bioética, Medicina do Trabalho e Medicina Física e Reabilitação, Faculdade de Medicina, Universidade de São Paulo (FMUSP), São Paulo, SP, Brazil; c Neuromodulation Center, Spaulding Rehabilitation Hospital, Harvard Medical School, Boston, MA, United States

**Keywords:** Amputation, Lower limbs, Timed up and go, Rehabilitation

## Abstract

The amputation of a limb directly impacts functioning. The lack of standardization in the assessment of lower-limb amputees complicates the identification of actual impairments. The Timed Up and Go (TUG) test has been validated and used in these individuals' clinical practice. The authors compared the performance of lower-limb amputees with that of healthy volunteers and identified the factors involved in performing the test and, consequently, in the patient´s functioning ability. Lower limb amputee individuals referred to the Institute of Physical Medicine and Rehabilitation of the Hospital das Clínicas of the Faculty of Medicine at the University of São Paulo (IMREA-HCFMUSP) were included, as well as healthy volunteers, between August 2020 and July 2022. Statistical data were analyzed using Stata 14, with a significance level set at α < 0.05. The results indicate that TUG times were statistically different between the groups, which can be explained by factors such as age, mobility measured by the Amputee Mobility Predictor Questionnaire (AMP), and prosthetic use. The TUG test can be standardized in clinical practice for amputee patients, and identifying the factors influencing its values supports the structuring of rehabilitation for lower-limb amputations.

## Highlights

• The timed Up and Go test (TUG) execution time is significantly slower in lower-limb amputees than in healthy volunteers.
• Older age, lower scores on the Amputee Mobility Predictor (AMP), and non-use of a prosthesis were key factors associated with slower TUG times.
• For each additional year of age, TUG time increased by 0.14 seconds in the amputee cohort.
• The TUG test is a practical tool that can be standardized in clinical practice to guide rehabilitation strategies for amputees.

## Introduction

Global epidemiological data indicate that approximately 35.3 million people have lower limb amputations.^1^ In Brazil, the lower limb amputation rate approached 24.4 per 100,000 inhabitants between 2010 and 2022, being considered a major public health concern.^2^

Amputation promotes functional, economic, and psychological implications and restrictions in social participation, in addition to requiring a long intervention process, from surgery to rehabilitation and inclusion of the individual into society.^2^^,^^3^ Appropriate assessment of the patient’s functioning and mobility status is necessary for their rehabilitation process. However, there is no current standardization of outcome measures for the clinical practice of lower limb amputees. To supply this need, the Consensus Outcome Measures for Prosthetic and Amputation Services (COMPASS) provided recommendations on such measurements.^4^

The TUG test was validated for the analysis of basic mobility, transfers, balance, and risk of falls in the amputee population.^5^^,^^6^ It corresponds to a test of simple and quick application, high intra-rater and inter-rater reliability, and high sensitivity and specificity.^7^ However, its use as a routine instrument for assessing physical mobility in these individuals and analyzing performance compared to non-amputees is still scarce in the literature.^6^

Therefore, the authors compared the TUG values of lower limb amputees with those of the non-amputee healthy volunteers from the DEFINE cohort study. The authors also identified which potential independent variables, including gender, age, amputation level, amputation time, etiology, use of walking aid device, and pain, could correlate with TUG measures in lower limb amputees.

## Materials and methods

This is a cross-sectional analysis, conducted in accordance with the STROBE Statement, and based on the baseline data of TUG in the amputee group of the DEFINE Cohort: Inhibitory deficit as a marker of neuroplasticity in rehabilitation^8^, approved by the Ethics Committee for the Analysis of Research Projects of HCFMUSP, under the registration CAAE: 86,832,518.7.0000.0068. All data used in this study are available in the text.

Amputees referred to the Instituto de Medicina Física e Reabilitação do Hospital das Clínicas da Faculdade de Medicina da Universidade de São Paulo (IMREA-HCFMUSP) and healthy controls were included in the study between August 2020 and July 2022. All participants signed the Informed Consent Form (ICF) and received an explanation about the processes and procedures performed, with the option of refusing to continue in cases of discomfort. A copy of the ICF was given to each participant.

The inclusion criteria were 18-years or older, with clinical stability confirmed by medical evaluation and the presence of lower limb amputation, regardless of the etiology. Exclusion criteria were pregnancy, history of stroke, traumatic brain injury, osteoarthritis, or any clinical or social condition that interferes with participation in rehabilitation.

Demographic data were collected from the medical records regarding age, gender, level, etiology, and time since amputation. Pain was assessed with the Visual Analogue Scale (VAS), a continuous scale of 100 mm horizontal lines anchored by two descriptors at each end. Patients were instructed to mark a line perpendicular to the scale, indicating their perceived pain intensity. A ruler measured the distance between the starting point and the patient's mark. This measurement yields a pain score ranging from zero to 10, with zero being “no pain” and 10 being “worst pain imaginable”.^9^

Mobility was determined by the Amputee Mobility Predictor Questionnaire (AMP), which includes questions about transfers, sitting and standing balance, and gait skills. The score for each item ranges from 0 to 2, where 0 indicates inability to perform the task, 1 implies that some assistance was required to complete the task, and 2 denotes complete independence. In 1995, in the United States, Medicare adopted a 5-level functional classification system (MFCL) to describe the functional abilities of people who have undergone lower limb amputation, based on the AMP (K0, K1, K2, K3, K4) that describes the ability or potential to use a prosthesis, and the likely prognosis of ambulation.^10^

Functional performance was assessed using the Timed Up and Go (TUG) test. Before the beginning of the test, a chair is positioned in an area free of obstacles or interruptions, and the patient is requested to be seated with his back against the chair. When the evaluator signals, the participant gets up from the chair, walks comfortably and safely for 3 m, rotates on his axis, and returns to the chair, repositioning his back in the starting position again.^11^ The time spent (in seconds) to perform the task is recorded. This test was performed three times, and the average of the three trials was used for analysis.^5^^,^^12^

Statistical analyses were performed to compare the TUG execution time between the amputee and control groups, as well as to test predictors of performance during the test among amputee patients. First, the data were tested for normality using the Shapiro-Wilk test and q-q plots for both the response variable (TUG) and the other continuous variables studied (age, injury time, and Amputee Mobility Predictor scale ‒ AMP). Considering the characteristics of the sample, the means of the TUG test between the groups were compared by *t*-test for unequal variances. Then, univariate linear regression analyses defined the variables correlated with the TUG test among amputee patients. Multiple linear regression analysis helped to determine the degree of association of the independent variables with the dependent variable, that is, how much the independent variables explain the changes of the dependent variable. Statistical tests were performed with Stata 14, and the established significance level was α < 0.05.

## Results

The authors included 63 participants with lower limb amputation and 115 healthy participants in the control group. A summary of the demographic and clinical data of these participants is shown in Table 1. The groups were observed to differ in terms of the proportion of men, average ages, years of education, BMI, and comorbidities.

**Table 1:** Baseline socioeconomic, clinical, and functional characteristics.

**Characteristics**	**Amputees** **(*n*=63)**	**Healthy volunteers** **(*n*=115)**
Men, n ( %)	52 (85.25 %)	38 (33.04 %)
Age, mean (± SD)	46.07 (15.23)	39.07 (12.12 %)
Years of schooling (± SD)	9.83 (3.25)	16.23 (3.77)
BMI (kg/m^2^), mean (± SD)	24.46 (5.97)	27.23 (5.02)
Low weight, n ( %)	11 (18.97 %)	1 (0.87 %)
Normal, n ( %)	28 (48.28 %)	48 (41.74 %)
Overweight, n ( %)	13 (22.41)	36 (31.30 %)
Obesity, n ( %)	6 (10.34 %)	30 (26.09 %)
Comorbidities, n ( %)		
Hypertension	22 (66.67 %)	11 (33.33 %)
Diabetes	25 (39.68 %)	3 (2.61 %)
Hypercholesterolemia	22 (34.92 %)	11 (9.57 %)
Months since amputation, mean (SD)	24.81 (19.12)	-
Etiology of amputation		
Traumatic, n ( %)	28 (44.44 %)	-
Vascular, n ( %)	24 (38.10 %)	-
Other, n ( %)	11 (17.46 %)	-
Affected side, n ( %)		
Left	36 (57.14 %)	-
Right	26 (41.27 %)	-
Bilateral	1 (1.59 %)	-
Level of amputation		
Choppart	1 (1.7 %)	-
Transtibial	22 (36.7 %)	-
Knee disarticulation	2 (3.3 %)	-
Transfemoral	32 (53.3 %)	-
Hip disarticulation	3 (5.0 %)	-
Use of prosthesis		
Yes	30 (47.6 %)	-
No	33 (52.4 %)	-
AMP score	32.57 (7.27)	-
AMP K0	0	-
AMP K1	5 (8.6 %)	-
AMP K2	19 (32.8 %)	-
AMP K3	25 (43.1 %)	-
AMP K4	9 (15.5 %)	-
Rehabilitation status		
Pre-Prosthetic Training	31 (49.21 %)	-
Prosthetic Training	23 (36.51 %)	-
Post-Prosthetic Training	7 (11.11 %)	-
Non-rehabilitated patients	2 (3.17 %)	-
TUG, seconds (SD)	19.67 (8.99)	8.39 (1.84) ***
TUG EMM	19.00 (0.76)**§**	8.72 (0.51)**§**
Assistive Technologies		
Crutches	48 (76.2 %)	-
Walker	7 (11.1 %)	-
None	8 (12.7 %)	-
VAS, cm (SD)	2.35 (1.74)	-

n, number; %, percentage; SD, standard deviation; BMI, Body mass Index; AMP, Amputee Mobility Predictor Questionnaire; TUG, Timed Up and Go; EEM, estimated marginal means; VAS, Visual Analog Scale; **§** Estimated marginal means described as means and standard error; *** *p* < 0.0001, Student *t*-test.

The normality tests showed that the TUG results of the control group are parametric and those of the amputee group are non-parametric. Among the others, the age of the amputee group and the AMP scale score were also considered parametric, while the other continuous variables were non-parametric. Considering the total sample size (*n* = 178), parametric tests were used.

TUG test times were statistically different between the groups (*p* < 0.0001). Considering possible imbalances between the cases and controls regarding sex, age, education, and comorbidities, post-hoc analyses comparing both groups were conducted with analysis of covariance (ANCOVA) such that sex, education, and presence of comorbidities were included as categorical covariables and age as a continuous variable of TUG. The results of ANCOVA evidenced that the TUG performance is influenced by age (*f* = 15.70; *p* < 0.001), whereas sex and presence of comorbidities did not significantly change the TUG performance in the comparison of the cases and controls (*f* = 2.14; *p* = 0.147 and *f* = 2.04; *p* = 0.155, respectively). Table 1 presents the observed means of TUG and marginal means of TUG considering the covariates analyzed by ANCOVA.

In the intra-group comparisons, considering the categorical variables of prosthetics, sex, level of amputation, and etiology, the results of the TUG were significantly different among patients with amputation of vascular etiology, as they performed worse in the TUG compared to those of amputation due to traumatic etiology (*p* = 0.0079), and between the groups formed by the use of assistive technologies, such as crutches and walker. Sex, use of prosthesis, level of amputation, and rehabilitation status did not generate different TUG performances, as shown in Table 2.

**Table 2:** Categorical variables associated with TUG.

**Variables**	**TUG**	**p-value**
Male	23.49 (8.46)	0.265 (t)
Female	18.94 (10.99)	
Prosthesis	19.26 (9.25)	0.731 (t)
No prosthesis	20.05 (8.87)	
Choppart	15.53 (-)	0.270 (a)
Transtibial	18.16 (7.51)	
Knee disarticulation	21.89 (4.66)	
Transfemoral	19.55 (10.33)	
Hip disarticulation	26.37 (5.52)	
Traumatic amputation	16.38 (7.79)	**0.0079** (t)
Vascular amputation	23.19 (9.56)	
Use of crutches	18.81 (7.70)	**<0.0001** (k)
Use of walkers	34.34 (5.60)	
No assistive technologies	12.06 (2.18)	
Pre-Prosthetic Training	18.85 (8.34)	0.885 (k)
Prosthetic Training	20.66 (9.92)	
Post-Prosthetic Training	19.30 (9.01)	
Non-rehabilitated patients	22.41 (14.55)	

t, test-T Student; a, ANOVA; k, Kruskal-Wallis.

Furthermore, the comparison between the groups that used or did not use Assistive Technologies was made using Kruskal-Wallis, as there was a considerable difference in the number of patients in these three groups. Therefore, for the regression analyses, the authors grouped the use of crutches and walkers (1) compared to no use (0), resulting in the fact that in the regressions, the use of assistive technology was not statistically significant in the final model, as shown in Table 3 below.

**Table 3:** Explanatory TUG regression models among patients with lower limb amputation.

	Univariate Regressions			
Variable	Coefficient	Confidence Interval		p-value
Age	0.31	0.19	0.44	**< 0.001**
BMI	0.30	−0.09	0.70	0.126
Years of schooling	−0.38	−1.09	0.32	0.280
Pain (VAS)	−0.74	−2.94	1.46	0.493
AMP classification	−6.52	−8.79	−4.24	**<0.001**
Time since amputation	−0.03	−0.15	0.09	0.616
				
	Final regression model (r² = 0.42; ***p*<0.0001**)			
Age	0.16	0.0	0.31	**0.039**
AMP classification	−4.71	−7.51	−1.91	**0.001**

BMI, Body Mass Index corrected by level of amputation; VAS, visual analog scale; AMP, Amputee Mobility Predictor Questionnaire.

Linear regression tests with the continuous variables age, BMI, years of education, pain, and AMP score demonstrated that, in isolation, the TUG test is influenced by age and the AMP classification. Therefore, considering the categorical and continuous variables concomitantly, a multiple linear regression model was generated with all the variables that were correlated with the time of execution of the TUG test, and from it, the model that best explains the result of this evaluation among patients with lower limb amputation, described in Table 3, was extracted.

## Discussion

TUG was statistically higher in the lower limb amputation group than in the healthy control group. The authors suggest that the higher execution time of lower limb amputees is explained by age, AMP score, and prosthesis use.

The values found in the amputee TUG differ from those previously by Wafi et al. (2023), who found an average of 30.6 ± 20.5 s in vascular amputees.^13^ In addition, an older study by Schoppen et al. (1999) obtained, for the elderly, an average of 24.5 s in the TUG.^14^ This value also diverges from the findings of Clemens et al. (2018) for men who have been traumatically amputated, who had an average of 10.1 s in transtibial amputees and 12.76 s in transfemoral amputees.^15^ These differences could be partially explained by the younger age of the patient population as well as the higher prevalence of traumatic etiology.

In fact, for amputee patients, advanced age increases the number of comorbidities that directly affect balance and mobility and consequently may increase the time of TUG.^16^ According to the present results, with each year of age, the TUG increased by 0.14 s. In addition, the inversely proportional relationship between MPA and TUG is in agreement with the findings by Sions et al. (2018), who obtained a mean TUG time of 12.82 s in K3 and a mean of 9.45 s in K4 patients.^17^ These results also demonstrate that the better the score on the AMP scale, the shorter the time it takes to perform the TUG test. They can be explained by the influence of the ability to balance and perform functional activities, measured in the items of the scale.^18^ All these relevant clinical findings will improve the care of the lower limb amputees and hopefully introduce TUG as a standardized functioning measurement for these patients.^16^

Amputation generates changes in gait patterns, including changes in the symmetry of joint forces and moments, as well as shorter stance phases and longer swing phases on the prosthetic side, reduced gait speed, and speed variation capacity.^19^ The modifications mentioned may explain the relationship between the use of the prosthesis and the time of the TUG. However, it is necessary to know if training with the technology occurred.

Most amputees use walking aids, at least during the early stages of adaptation.^19^ Using assistive devices, such as canes or walkers, increases stability, provides support, and prevents falls, in addition to favoring or reducing the weight bearing under the prosthesis, corresponding to an essential phase of gait. However, it may increase the time required to perform the TUG due to reduced mobility.^14^ Finally, although the pain variable does not have a significant impact on the TUG, it is estimated that two-thirds of lower limb amputees suffer from chronic pain, creating a complex obstacle that is difficult to manage and has a meaningful functional impact.^1^

The TUG test is a simple, fast, and reliable assessment of functional capacity in amputee patients. Few studies, if any, were conducted in Latin American countries to capture TUG values in amputee patients. Future amputee rehabilitation programs should consider faster and more effective prosthesis use and include functional items in AMP assessments.

TUG assessments are valuable for assessing the functional status of amputee patients, irrespective of etiology. The influence of age, prosthetic use, and AMP scores suggests the relevance of faster and quality rehabilitation services provision. Especially in countries like Brazil, prosthetic use may take a long time to occur. This delay may hinder the functional ability of these patients. The present study suggests that TUG can be used systematically in amputee patients. TUG values should be collected in other settings to better understand the potential factors influencing its values and the generalizability of these findings. Precise detection and understanding of these related variables will certainly improve the rehabilitation provision of lower limb amputations.

Although the results of this study provide relevant information, it is important to acknowledge some limitations. Firstly, the heterogeneity of the sample groups (amputees and healthy volunteers) and the intragroup differences, such as whether or not prior rehabilitation was performed before the test assessment of unilateral or bilateral amputations, may affect the generalization of the results. The authors must also consider that the TUG does not assess qualitative aspects such as prosthetic weight-bearing, proper postural alignment, correct use of assistive technologies, and safety during the steps, especially during the turn. Therefore, it cannot be claimed that there is a better or worse gait quality, or which functional tasks are included in the test are affected.

## Conclusion

The TUG values of lower limb amputees are statistically higher than those of healthy volunteers. Age, AMP scores, and the use of prostheses are associated with TUG measures in lower limb amputees. The level of amputation, duration, etiology, use of mobility aids, and pain do not influence TUG values in lower limb amputees.

## Data availability

The datasets generated and/or analyzed during the current study are available from the corresponding author upon reasonable request.

## Declaration of competing interest

The authors declare no conflicts of interest.
